# Imaging findings of uterine adenosarcoma with sarcomatous overgrowth: two case reports, emphasizing restricted diffusion on diffusion weighted imaging

**DOI:** 10.1186/s12905-021-01567-z

**Published:** 2021-12-16

**Authors:** Go Nakai, Hiroki Matsutani, Takashi Yamada, Masahide Ohmichi, Kazuhiro Yamamoto, Keigo Osuga

**Affiliations:** 1 The Department of Diagnostic Radiology, Osaka Medical and Pharmaceutical University, 2-7 Daigaku-machi, Takatsuki City, Osaka 569-8686 Japan; 2 The Department of Pathology, Osaka Medical and Pharmaceutical University, 2-7 Daigaku-machi, Takatsuki City, Osaka 569-8686 Japan; 3 The Department of Obstetrics and Gynecology, Osaka Medical and Pharmaceutical University, 2-7 Daigaku-machi, Takatsuki City, Osaka 569-8686 Japan

**Keywords:** Magnetic resonance imaging, Adenosarcoma, Sarcomatous overgrowth, Diffusion weighted imaging, Case report

## Abstract

**Background:**

Adenosarcoma is classified as a mixed epithelial and mesenchymal tumor composed of a benign epithelial component and a malignant stromal component. The stromal component in adenosarcoma is usually low grade, and consequently the prognosis is relatively favorable. While, adenosarcoma with sarcomatous overgrowth (SO) is defined as an adenosarcoma in which the sarcomatous component constitutes more than 25% of the tumor. The stromal component is also high-grade sarcoma showing greater nuclear pleomorphism and mitotic activity, thus, it is associated with worse prognosis. MRI findings of adenosarcoma without SO have been described in previous literatures but the imaging findings in adenosarcoma with SO may be poorly defined. Therefore we present two cases of uterine adenosarcoma with SO.

**Case presentation:**

Patient 1 was a 76-year-old woman referred to our hospital with complaint of abdominal distension and postmenopausal bleeding. Patient 2 was a 57-year-old woman with complaint of lower abdominal pain and abnormal uterine bleeding. On magnetic resonance imaging (MRI), T2 weighted imaging showed a large, heterogeneous high-intensity mass with hyperintense tiny cysts that expanded the uterine cavity and extended into the cervical canal for both patients. On diffusion-weighted imaging (DWI), both masses appeared as high signal intensity. Patient 2 also had a right ovarian adult granulosa cell tumor that may have contributed to development of the adenosarcoma. Patient 1 recurred with peritoneal sarcomatosis 6 months after surgery and died of the disease. Patient 2 also recurred with a left upper lung metastasis 3 months after surgery.

**Conclusions:**

DWI may depict pathological changes produced by SO of adenosarcoma as high signal intensity, even though SO does not seem to change MRI findings of adenosarcoma on other sequences. Therefore, DWI could potentially predict SO in presumptive adenosarcoma on MRI and the patient’s prognosis. It is also important for pathologists to know if SO can arise in adenosarcoma because they need to examine the tumor thoroughly to determine the percentage of SO component in the tumor volume when SO is present.

## Background

Adenosarcoma is classified as a mixed epithelial and mesenchymal tumor composed of a benign epithelial component and a malignant stromal component. The stromal component in adenosarcoma is usually low grade, and consequently the prognosis is relatively favorable compared with that of other gynecological sarcomas [1]. However, sarcomatous overgrowth (SO), in which the sarcomatous component showing greater nuclear pleomorphism and mitotic activity constitutes more than 25% of the tumor [2], often results in myometrial invasion and vascular invasion, leading to a worse prognosis [3]. Although MRI findings of adenosarcoma without SO have been described in the literature [4, 5], the imaging findings in adenosarcoma with SO may be poorly defined. [6].

## Case presentation

### Case 1

A 76-year-old woman (para [P] 1) referred to our hospital with a chief complaint of abdominal distension and postmenopausal bleeding for 4 months consulted a gynecologist and was found to have a bulky uterus with a 132-mm subendometrial mass on vaginal ultrasonography. Adenosarcoma was suspected on the basis of findings from biopsy by hysteroscopy. She had no significant past medical history. Serum cancer antigen (CA) 125 and CA 19–9 were 283 U/ml (normal range 0–35 U/ml) and 11.5 U/ml (normal range 0–35 U/ml) respectively.

**Fig. 1:**
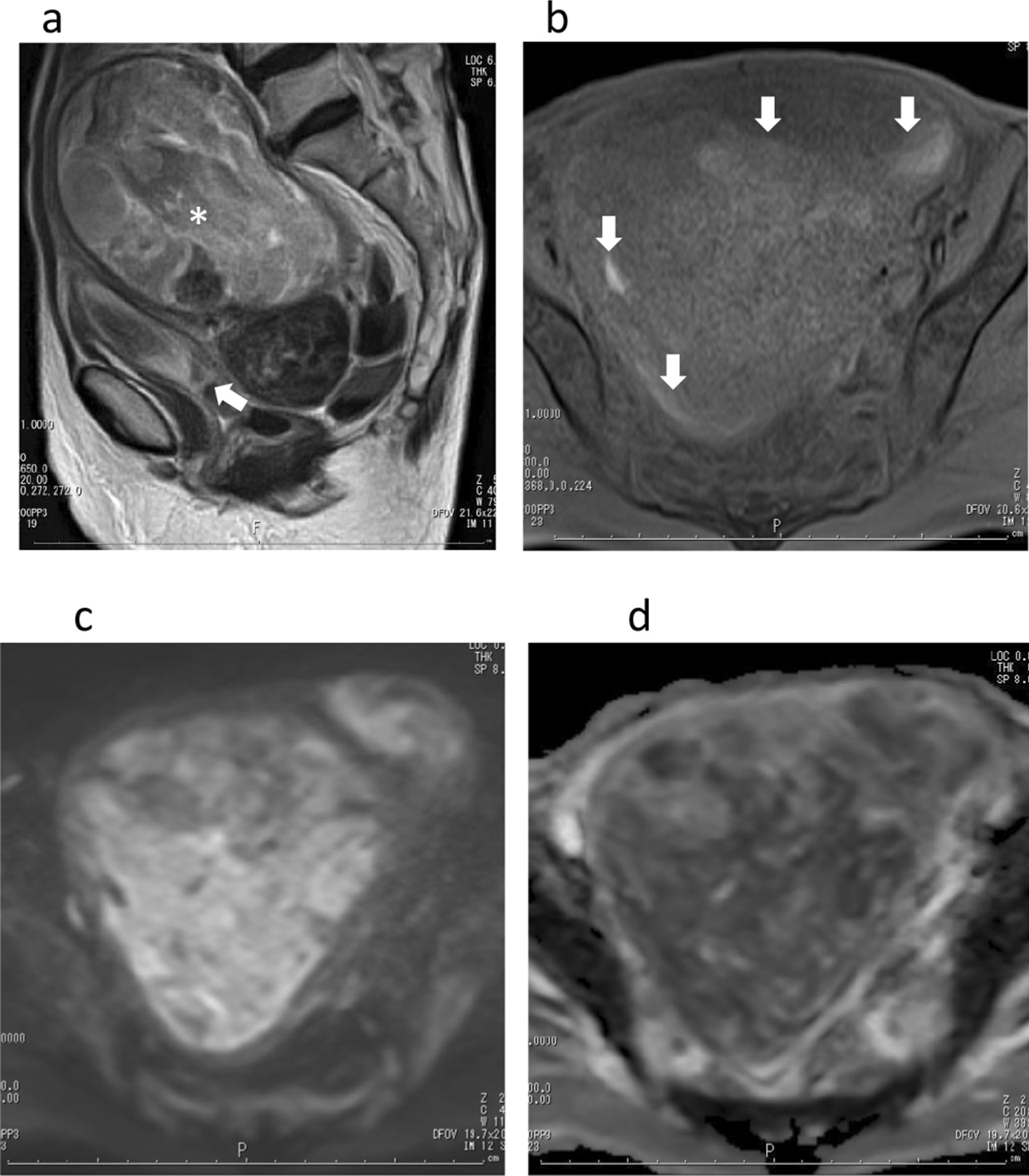
(**a**) Sagittal T2-weighted imaging (repetition time [TR]/echo time [TE], 4650/120 ms) showed a large, heterogeneous high-intensity mass without myometrial invasion expanding the uterine cavity (*) and extending into the cervical canal (arrow). (**b**) The mass showed low signal intensity with areas of slightly high signal intensity (arrows) on fat-suppressed T1-weighted imaging (TR/TE, 600/10 ms). (**c**) The mass showed high intensity on diffusion-weighted imaging (b = 1000 s/mm^2^, TR/TE, 7000/100 ms) and low intensity (1.26 × 10^−3^ mm^2^/s) on apparent diffusion coefficient maps (**d**)

Magnetic resonance imaging (MRI) was performed at 1.5 Tesra for further examination. Sagittal T2-weighted imaging (WI) (repetition time [TR]/echo time [TE], 4650/120 ms) showed a large, heterogeneous high-intensity mass, approximately 241 × 114 × 88 mm^3^ in size, without myometrial invasion expanding the uterine cavity and extending into the cervical canal (Fig. 1a). The enlarged uterine size was 250 × 126 × 99 mm^3^. The mass showed low intensity with areas of slightly high signal intensity on fat-suppressed T1WI (TR/TE, 600/10 ms) (Fig. 1b). The mass showed inhomogeneous contrast enhancement with cystic changes of variable sizes and necrotic foci, high intensity on diffusion-weighted imaging (DWI) (b = 1000 s/mm^2^, TR/TE, 7000/100 ms) (Fig. 1c) and low intensity (1.26 × 10^−3^ mm^2^/s) on apparent diffusion coefficient (ADC) maps. Uterine carcinosarcoma or adenosarcoma was suspected as the preoperative diagnosis.

Total abdominal hysterectomy and bilateral adnexectomy with pelvic lymphadenectomy were subsequently performed.

Benign glandular epithelial components surrounded by atypical stromal cells with a high mitotic rate (20/10 high power field [HPF]) were identified on pathological examination. High-intensity areas observed on T1WI corresponded to hemorrhage, and SO was present. The tumor was diagnosed as adenosarcoma with SO without myometrial invasion (T1aN0M0).

A follow-up CT scan performed 6 months after surgery revealed peritoneal sarcomatosis. Although her gynecologist recommended adjuvant chemotherapy, she did not wish to receive the treatment and died of the disease 1.5 months after recurrence.

### Case 2

A 57-year-old woman (P 3) with complaint of low abdominal pain and abnormal uterine bleeding for 3 months consulted a gynecologist. Menstrual cycle length ranged from 30 to 40 days. She had no significant past medical history. Endometrial biopsy indicated atypical endometrial hyperplasia. Vaginal ultrasonography showed an enlarged uterus measuring 150 mm accompanied by a 73-mm tumor in the anterior wall, and both ovaries were not visualized. CA 125 and CA 19–9 were 40.3 U/ml (normal range 0–35 U/ml) and 1.1 U/ml (normal range 0–35 U/ml), respectively. Serum estradiol (E2) was within the normal limit (130.0 pg/mL).

**Fig. 2:**
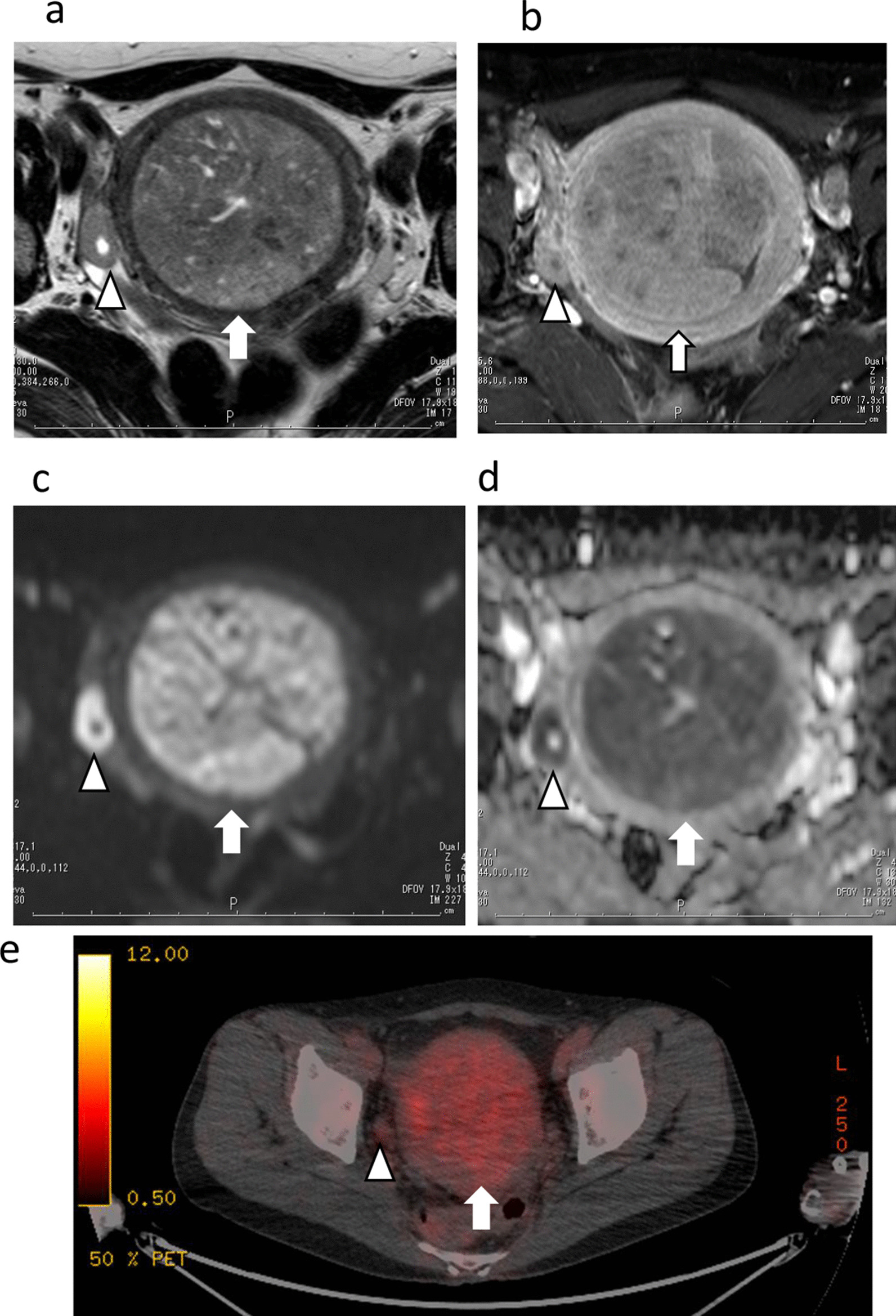
(**a**) Axial T2 weighted imaging (TR/TE, 6130/100 ms) showed an intrauterine mass (arrow) with clearly defined inhomogeneous high intensity. (**b**) The mass (arrow) showed inhomogeneous contrast enhancement with cystic changes of variable sizes on post-contrast fat-suppressed T1 weighted imaging (TR/TE, 575/13 ms), high signal intensity on diffusion-weighted imaging (DWI) (b = 1000 s/mm^2^, TR/TE, 4317/70 ms) (**c**) and low signal intensity (0.88 × 10^−3^ mm^2^/s) on apparent diffusion coefficient (ADC) maps (**d**). A 22-mm solid nodule accompanied by a tiny cyst was detected in the right ovary (**a**–**d** arrowhead). The nodule showed homogenous iso signal intensity with the uterine tumor on T2WI (**a**). It showed homogeneous contrast enhancement with a tiny cystic change (**b**), marked high signal intensity on DWI (**c**) and low signal intensity (0.66 × 10^−3^ mm^2^/s) on ADC maps (**d**). Whole-body 18F-FDG positron emission tomography-computed tomography (PET-CT) showed increased FDG uptake (maximum standardized uptake value: 8.2) in the uterine tumor (arrow) and no abnormal FDG uptake in any other organs, including the right ovarian nodule (**e** arrowhead)

MRI showed a intrauterine mass, approximately 96 × 74 × 57 mm^3^ in size, protruding into the cervical canal with clearly defined inhomogeneous high intensity on T2WI (TR/TE, 6130/100 ms) (Fig. 2a) and low intensity with a slight high signal intensity area on fat-suppressed T1WI (TR/TE, 575/13 ms). The enlarged uterine size was 129 × 82 × 76 mm^3^.The mass showed inhomogeneous contrast enhancement with cystic changes of variable sizes and necrotic foci (Fig. 2b), high intensity on DWI (b = 1000 s/mm^2^, TR/TE, 4317/70 ms) (Fig. 2c) and low intensity (0.88 × 10^−3^ mm^2^/s) on ADC maps (Fig. 2d). A 22-mm solid nodule accompanied by a tiny cyst was detected in the right ovary. T2WI showed homogenous iso signal intensity with the uterine tumor on T2WI (Fig. 2a) and low intensity on T1WI. The nodule had homogeneous contrast enhancement with a tiny cystic change (Fig. 2b), high intensity on DWI (Fig. 2c), and low intensity (0.66 × 10^−3^ mm^2^/s) on ADC maps (Fig. 2d).

Whole-body 18F-fluorodeoxyglucose (FDG) positron emission tomography-computed tomography (PET-CT) showed increased FDG uptake (maximum standardized uptake value: 8.2) in the uterine tumor and no abnormal FDG uptake in any other organs, including the right ovarian nodule (Fig. 2e). Uterine carcinosarcoma or adenosarcoma metastasizing to the right ovary was suspected as the preoperative diagnosis.

The patient underwent abdominal modified radical hysterectomy with bilateral adnexectomy, pelvic and para-aortic lymphadenectomy, and omentectomy.

Benign glandular epithelial components surrounded by atypical stromal cells with a high mitotic rate (15/10 HPF) with minimal myometrial invasion and SO were present in the uterine tumor on pathological examination, and thus the tumor was diagnosed as adenosarcoma with SO (T1bN0M0). The right ovarian nodule was diagnosed as an adult granulosa cell tumor (GCT).

A follow-up CT scan revealed a left upper lung metastasis 3 months after surgery. Accordingly, the patient underwent thoracoscopic segmentectomy of the left upper lobe. As of the writing of this report, at 22 months after the surgery, she has showed no signs of recurrence.

## Discussion and conclusions

Adenosarcoma with SO is defined as an adenosarcoma in which the sarcomatous component constitutes more than 25% of the tumor [2]. The stromal component is also high-grade sarcoma showing greater nuclear pleomorphism and mitotic activity, leading to a worse prognosis. Recurrence was observed within 6 months after surgery in both of our patients and one died of the disease, probably due to its aggressive clinical behavior and disease progression.

MRI findings of adenosarcoma have been previously described in the literature [4, 5, 7]. It typically presents as a solitary, exophytic, polypoid mass in the endometrial cavity that protrudes through the cervical os. The mass consists of heterogeneous solid components with tiny cysts corresponding to glandular cavities that show high signal intensity on T2WI. The solid component shows heterogenous high signal intensity compared to the myometrium on T2WI and low- or iso-intensity compared to the myometrium on gadolinium-enhanced T1WI. Hemorrhagic necrosis showing high signal intensity on T1WI is common, particularly when SO is present. These MRI findings are common irrespective of the presence of SO, and are consistent with MRI findings in our patients. However, only two previous reports have described DWI findings of adenosarcoma. Fujii et al. reported a case of adenosarcoma without SO arising from adenomyosis showing slightly high signal intensity on DWI and high intensity (1.71 × 10^−3^ mm^2^/s) on ADC maps. Benign glandular epithelial components surrounded by atypical stromal cells with mitosis (2–3/10 HPF) were identified on pathological examination [7]. Takeuchi et al. reported uterine low-grade adenosarcoma without a marked signal increase throughout the tumor on DWI. As this contrasts with the marked increase in signal on DWI that we observed in our patients who had adenosarcoma with SO, we assume the presence of SO is associated with increasing signal intensity on DWI.

The diagnosis of adenofibroma was removed from the WHO Classification of Tumours, 5th Edition, Volume 4 [8], and it is believed that the majority of tumors previously diagnosed as adenofibroma were simply low-grade adenosarcomas or endometrial or cervical polyps with unusual morphology. Endometrial polyps usually show relatively lower signal intensity than malignant endometrial tumors on DWI [9] because they are composed of irregularly shaped glands with fibrous stroma, resulting in lower cellularity than highly cellular malignant tumors. The stromal component in adenosarcoma is usually low grade, and thus uterine cervical adenosarcoma initially diagnosed as a cervical polyp pathologically can present as a recurrent cervical polyp [10]. Consequently, it may occasionally be challenging to differentiate adenosarcoma from polyps even pathologically, as polyps may have cellular stroma and architectural features that overlap to some degree with those of adenosarcoma [11]. Given these similarities in pathologic features between adenosarcomas and polyps, the reported signal intensity of adenosarcoma without SO on DWI is consistent with its pathological features, whereas SO showing greater nuclear pleomorphism and mitotic activity might result in markedly high signal intensity on DWI as seen in our patients. Therefore, DWI could potentially be used to predict SO in presumptive adenosarcoma on MRI and the patient’s prognosis. It is also important for pathologists to know if SO can arise in adenosarcoma because they need to examine the tumor thoroughly to determine the percentage of SO component in the tumor volume when SO is present.

Although tumor size is not regarded as important in the diagnosis of adenosarcoma, it is important to note that mean size of polyps (1.4–2.5 cm) is much smaller than that of adenosarcoma (6.5 cm) [9, 11]. Large tumor size (major axis > 10 cm) is not a good indicator of SO either because many tumors without SO are also large [4, 5, 12], although high mitotic rate seems to be associated with large tumor size.

FDG PET-CT findings in uterine adenosarcoma have never been reported. However, there has been one report of FDG PET-CT findings of recurrent tumors in a patient with uterine cervical adenosarcoma with SO [13]. According to that report, recurrent tumors in the vaginal vault, right pelvic lymph nodes and pubic ramus showed intense FDG uptake, although preoperative PET-CT was not performed. Given the intense FDG uptake in the primary lesion in Patient 2 and the metastatic lesions in the previous report, the right ovarian nodule in Patient 2 which showed high signal intensity on DWI without any FDG uptake on PET-CT should not have been regarded as a metastasis. According to previous reports describing imaging findings of GCTs, they showed mild FDG uptake on PET-CT and restricted diffusion (high signal intensity) on DWI [14, 15], although FDG uptake of GCT may vary widely due to its variety of clinical features and uncertain potential of malignancy [16, 17]. In Patient 2, E2 continuously produced by the right ovarian GCT might be one of the causes of the uterine adenosarcoma with SO because studies have shown an association with long-term unopposed oestrogen therapy such as tamoxifen therapy [12, 18].

In conclusion, DWI may depict pathological changes produced by SO of adenosarcoma as high signal intensity, even though SO does not seem to change MRI findings of adenosarcoma on other sequences. Therefore, DWI could potentially predict SO in presumptive adenosarcoma on MRI and the patient’s prognosis. It is also important for pathologists to know if SO can arise in adenosarcoma because they need to examine the tumor thoroughly to determine the percentage of SO component in the tumor volume when SO is present.

## Data Availability

The datasets used and/or analyzed during the current study are available from the corresponding author on reasonable request.

## Abbreviations

SO: Sarcomatous overgrowth
G: Gravida
P: Para
CA: Cancer antigen
MRI: Magnetic resonance imaging
WI: Weighted imaging
TR: Repetition time
TE: Echo time
DWI: Diffusion-weighted imaging
ADC: Apparent diffusion coefficient
HPF: High power field
E2: Estradiol
FDG: Fluorodeoxyglucose
PET-CT: Positron emission tomography-computed tomography
GCT: Adult granulosa cell tumor
